# Characterization of aroma profile and microbial community of cigar tobacco leaves from different aging periods and varieties and their correlations analysis

**DOI:** 10.1186/s40643-025-00906-4

**Published:** 2025-07-28

**Authors:** Jian Liu, Zelin He, Qing Lin, Can Lyu, Junwei Zhao, Xiang Li, Xueying Wang, Yansong Xiao, Yang Ning

**Affiliations:** 1 Beijing Life Science Academy, Beijing, 102209 China; 2 Hunan Tobacco Company Chenzhou Company, Chenzhou, 423001 China; 3 Institute of Tobacco Research, Chinese Academy of Agricultural Sciences, Qingdao, 266101 China; 4 Fujian Tobacco Company Nanping Company, Nanping, 353000 China; 5 Anhui Wannan Tobacco Leaf Company Limited, Xuancheng, 242000 China

**Keywords:** Cigar, Microbial community, Aroma, Aging period, Variety

## Abstract

In this study, the CTLs from Hainan, China, were used, the aroma profiles of CTLs from five years (2017–2021) and three varieties [Hainan2 (HN2), Jianheng3 (JH3), and Guyin4 (GY4)] were analyzed, and the microbial communities of these CTLs and the relation with aroma constituents were explored. Results revealed the contents of total aroma constituents (3.55–7.67 mg/kg), esters (47.4–824 μg/kg), alcohols (123–561 μg/kg), aldehydes (51.4–97.1 μg/kg), and nitrogen heterocycles (1.86–4.25 mg/kg), firstly increased and subsequently declined with the increase of years, whereas ketones (1.07–3.56 mg/kg) exhibited an inverse trend. Among the different varieties, the highest aldehyde (142 μg/kg) content was observed in JH3, while the highest total aroma constituents, acids, ketones, and nitrogen heterocycles were found in GY4, reaching 10.5, 0.604, 1.86, 6.43 mg/kg, respectively. Furthermore, the bacterial communities of CTLs from different years exhibited discernible succession with increasing years. The abundances of *Staphylococcus* (3.03–47.5%) and *Sphingomonas* (1.18–10.2%) first increased and then decreased, whereas *Pseudomonas* (1.63–11.5%), *Ralstonia* (0.669–20.2%), and *Pantoea* (6.07–10.5%) exhibited the opposite trend, and *Aspergillus* and *Cladosporium* with total abundances of 90.2–94.2% were predominant in all years. The dominant bacteria and fungi of CTLs from different varieties were identified as *Staphylococcus* (15.0–81.1%) and *Aspergillus* (45.6–85.0%), respectively. However, the dominant microbial abundances in JH3 were significantly lower than those in HN2 and GY4, while other microorganism abundances were increased. Partial least square-regression analysis demonstrated that *Paracoccus*, *Staphylococcus*, *Aerococcus*, *Pantoea*, *Methylobacterium*-*Methylorubrum*, and *Bacillus* of CTLs from different years were associated with aroma constituents.

**Graphical Abstract:**

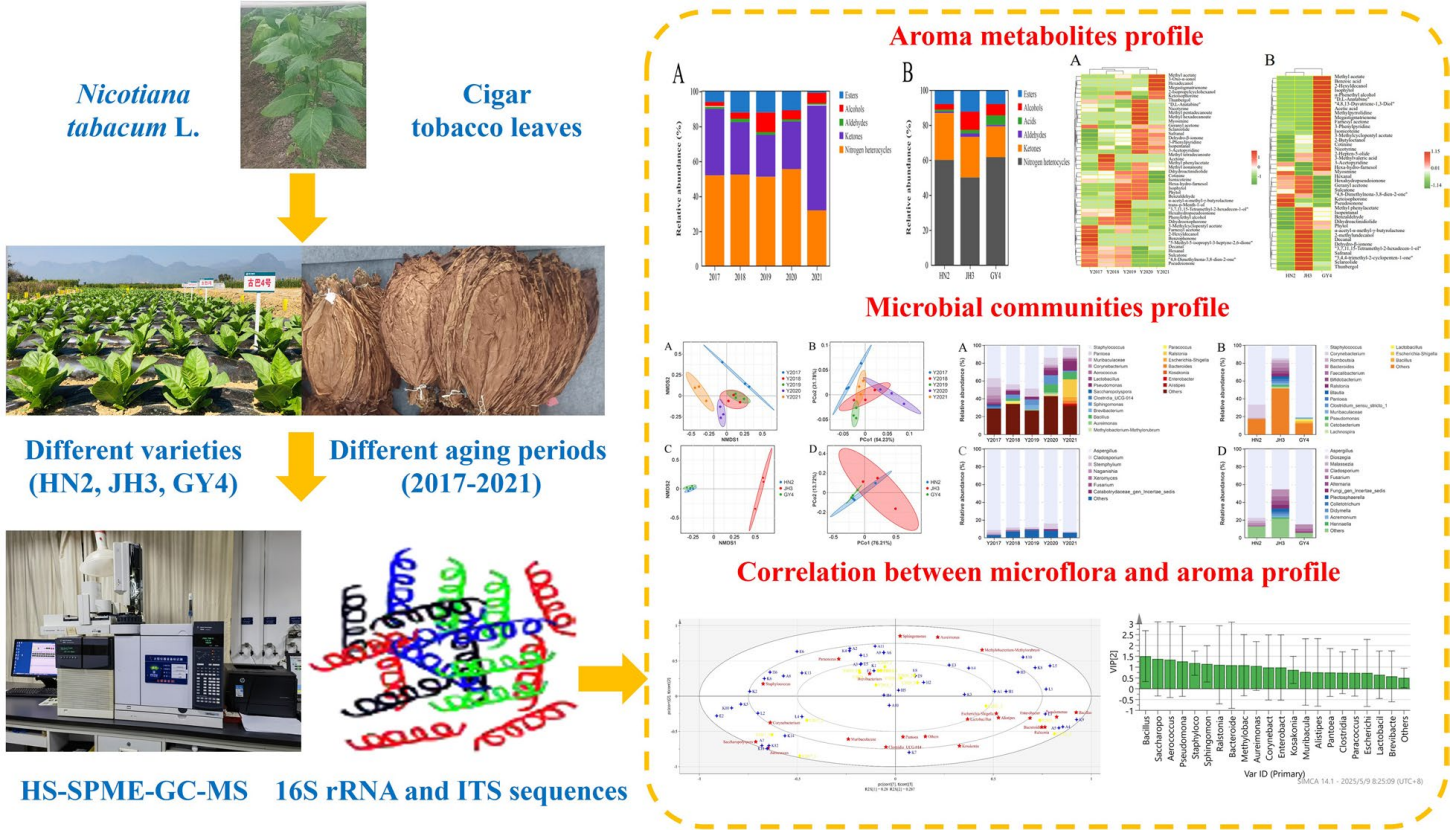

**Supplementary Information:**

The online version contains supplementary material available at 10.1186/s40643-025-00906-4.

## Introduction

The cigar is famous for its long history, and its flavor is strong, mellow, and varied compared with flue-cured tobacco, which includes fruity, floral, nutty, chocolate, coffee, and milk aromas (Morris and Fiala 2015). Flavor is the core of cigar tobacco leaves (CTLs), it determines quality characteristics (Shi et al. 2023), and the compositions, contents, and balance of chemical constituents are the basis for determining flavor profile, and the chemical constituent profiles are affected by variety, planting environment, and production technology, etc.

The elaboration of cigar includes the cultivation, air-curing, fermentation, rolling, and aging, which together with the accumulation, degradation, and transformation of macromolecular, and the corresponding biosynthesis of aroma precursors and volatile flavor compounds, and process is related to microorganisms (Wu et al. 2023a). For example, the microbial community of CTLs from Dominica, Brazil, Indonesia, and China was related to the most volatile flavor compounds (Zheng et al. 2022). Bacteria take part in metabolism of sugar, lipid, and amino acids, including *Staphylococcus*, *Pseudomonas*, *Bacillus*, *Brevundimonas*, *Aerococcus*, *Lactobacillus*, and fungal decomposition of lignin, cellulose, and pectin by saprophytism during air-curing and fermentation (Zhang et al. 2023).

The aging of cigar results in a better appearance and flavor and reduces pungent odors, nicotine, and unpleasant sensations of smoke such as roughness, pungency, and bitterness, as the sensory characteristics are improved by a more balanced chemical constituent. Degradation products of cedrans, phenylalanines, and carotenoids were significantly increased during the spontaneous aging of flue-cured tobacco, browning reaction products were almost unchanged, and neoprenoid content was slightly decreased (Song 2015). Predominant bacteria included forty-eight genera and *Bacillus* was predominant in all aging flue-cured tobacco (Wang et al. 2018). However, spontaneous aging is less efficient as a time-consuming process, the reveal of microbial community and flavor profile contributes to exploiting the core microflora to speed or reform the traditional spontaneous aging (Qi et al. 2018). Besides, breeding and selection of fine varieties are the prerequisites for obtaining tobacco leaves with an outstanding aroma profile (Xian et al. 1992). For the tobacco varieties with obvious defects in aroma profile, it is difficult to compensate for these defects through cultivation measures (Liu 2013). Biological factors can significantly affect the content of plant metabolites, but there are few reports on the regulation of cigar flavor from the perspective of varieties.

The different microbial community functions are mainly associated with alterations in communities during fermentation, and the desired community functions could be obtained based on the optimization or design of the microbial communities. The contribution of microorganisms to the aroma formation was well documented, as well as the effects of varieties, origins, process parameters, and microbial agents on the microbial communities and aroma profiles during the air-curing and fermentation for production. For example, *Phoma*, *Mycosphaerella*, *Wallemia*, and *Cladosporium* of fungi affected the flavor, while *Pantoea* and *Staphylococcus* were positively correlated with phenylacetaldehyde and acetophenone during the air-curing (Zhang et al. 2025). *Trichosporon*, *Thioalkalicoccus*, and *Jeotgalicoccus* during the fermentation were associated with tetramethylpyrazin, geranyl acetone, and hexahydropseudoionone, respectively (Wu et al. 2023a). Fungal genera *Golubevia*, *Bulleromyces*, *Aspergillus*, *Candida*, and bacteria including *Brevundimonas*, *Corynebacterium*, *Massilia* were correlated with flavor constituents of fermented CTLs by O2PLS analysis (Zhang et al. 2024). The dominant microorganisms may not directly contribute to the flavor formation, but they affect the community composition and abundance and other microorganisms through community interactions, thus the flavor compounds are modified. Bacteria of cigar strongly correlate with aldehyde and ketone than fungi, which enhances flavor constituents and overall quality (Zhang et al. 2024).

Correlation analysis contributes to identifying functional microorganisms and advancing achievement of microbial agents. For instance, Wu et al. (2022) reported that *Aspergillus* was correlated with the production of flavor constituents, the found *Aspergillus nidulans* F4 contributed to decomposition in fatty acid and terpenoid. In vitro isolation and bioaugmentation with *Candida parapsilosis* and *Candida metapsilosis* have been demonstrated to significantly reduce alkaloid in tobacco leaves, and increase the flavor constituents (Jia et al. 2023). Additionally, the incorporation of *Bacillus altitudinis* has been found to enhance the transformation of macromolecules in CTLs and effectively promote aroma production, resulting in a 43% increase in total aroma production compared to natural fermentation (Song et al. 2024).

The influences of production year from five years and the raw material from three varieties on the aroma profile of CTLs were analyzed in this study. The aroma constituents of these CTLs and discrepancies in microbial community diversity and structure were explored. Relationship between dominant microorganisms and volatiles was also revealed using multivariate statistical analysis. The exploration of the function of microorganisms in different CTLs of aging periods and varieties lays a foundation for the efficient regulation of the aroma quality of cigar.

## Materials and methods

### Materials and reagents

CTLs were acquired from the production regions in Hainan, China (Table 1). The reagents were obtained from Sinopharm Chemical Reagent Co., Ltd (Shanghai, China). The phenethyl acetate standard was obtained from Anpel Laboratory Technologies Inc. (Shanghai, China).

**Table 1:** The cigar tobacco leaves used in this study

No	Origin	Year	Variety	Leaf part
1	Guangcun	2017	Hainan2	Middle
2	Guangcun	2018	Hainan2	Middle
3	Guangcun	2019	Hainan2	Middle
4	Guangcun	2020	Hainan2	Middle
5	Guangcun	2021	Hainan2	Middle
6	Changjiang	2020	Hainan2	Middle
7	Changjiang	2020	Jianheng3	Middle
8	Changjiang	2020	Guyin4	Middle

### Analysis of aroma constituents

The aroma constituents of CTLs were analyzed by HS–SPME–GC–MS, based on report of Wu et al. (2023b). 0.50 g of CTL powders were weighed and mixed with 2.00 μL of 105 mg/L phenethyl acetate in a vial. The vial was rapidly sealed and shaken. Then mixture was heated at 70.0 °C for 30.0 min, and it was extracted by a 50/30 μm DVB/CAR/PDMS fiber (Supelco Inc., Bellefonte, PA, USA) for 30.0 min.

GC–MS (7890A/5977C, Agilent Technologies Inc., Santa Clara, CA, USA) equipped with an elastomeric quartz capillary column (DB-5MS, 30 m × 0.25 mm × 0.25 μm, Agilent Technologies Inc.) was selected to perform the analysis. Heating program was as follows: the initial temperature was 40.0 °C and held on for 2.00 min, it increased to 220 °C at a speed of 6.00 °C/min and held on for 0.00 min, then elevated to 280 °C at a speed of 20.0 °C/min and maintained for 10.0 min, the carrier gas was He (99.999%) and injection port temperature was 240 °C. The conditions of mass spectrometry were as follows: the transmission line and ion source temperatures were 290 °C and 230 °C, respectively, EI with 70 eV ionization voltage, and full scanning with 33–325 amu. The aroma constituents were qualified by comparative analysis of the mass spectrometry according to the NIST 17 standard library, and they were semi-quantified by comparing the area of total ion chromatogram with phenethyl acetate.

### Analysis of microbial communities

The DNAs (n = 3) of microorganisms in samples were extracted by CTAB or SDS methods, after which the DNA purity was analyzed by agarose gel electrophoresis, and concentration was adjusted to 1 ng/μL. Based on diluted DNA, 16SV34 region and ITS1 region were selected as the sequencing regions, and the primers of 341F and 806R, ITS1-F and ITS2 were used for PCR amplification. The library construction kit was utilized to construct the library. After Qubit and q-PCR quantification, the constructed libraries were quantified and then sequenced by NovaSeq 6000 PE250.

Effective tags were obtained by splicing the reads of each sample using FLASH, strict filtering, and a quality control process of QIIME’s tags. The entire effective tags were clustered by Uparse, and the sequences were clustered into OTUs at 97% similarity. The annotation of species was carried out by Mothur method and SSU rRNA database.

### Statistical analysis

All the analysis was repeated three times, and the experimental results were presented as mean ± standard deviation. IBM SPSS Statistics 25 (IBM Corp., Armonk, NY, USA) was used to perform a one-way analysis of variance (ANOVA) and Duncan’s multiple comparison test to evaluate the differences between samples (Zhao and Zhao 2022). Partial least square regression (PLSR) was carried out using Simca 14.1 (Mks Umetrics Ab Corp., Malmö, Sweden) to correlate aroma constituents with microorganisms. OmicShare tool (https://www.omicshare.com/tools) was used to perform the non-metric multidimensional scaling (NMDS) analysis and principal coordinate analysis (PCoA).

## Results and discussion

### The aroma profile and differential constituent of CTLs from different aging periods

A total of 46 aroma constituents were detected in CTLs over the 5 years, they were divided into five classes, which included ketones (14), alcohols (11), esters (10), nitrogen heterocycles (6), and aldehydes (5), with the contents of 1.07–3.56 mg/kg, 123–561 μg/kg, 47.4–824 μg/kg, 1.86–4.25 mg/kg, and 51.4–97.1 μg/kg, respectively, and the total aroma constituent contents ranging from 3.55 to 7.67 mg/kg (Table S1). There were significant differences in the aroma constituent contents in different CTLs. The contents of esters, alcohols, aldehydes, and nitrogen heterocycles in CTLs increased first and then decreased with increasing years, while the ketones decreased with increasing years from 2018 to 2021. Among them, the contents of alcohols and aldehydes in 2019 CTL increased significantly compared to other years, by 0.317–3.57 and 0.236–0.888 times, respectively, the esters and nitrogen heterocycles increased in 2020 by 0.365–16.4 and 0.655–1.28 times, respectively, and ketones increased in 2021 by 0.702–2.33 times.

**Fig. 1:**
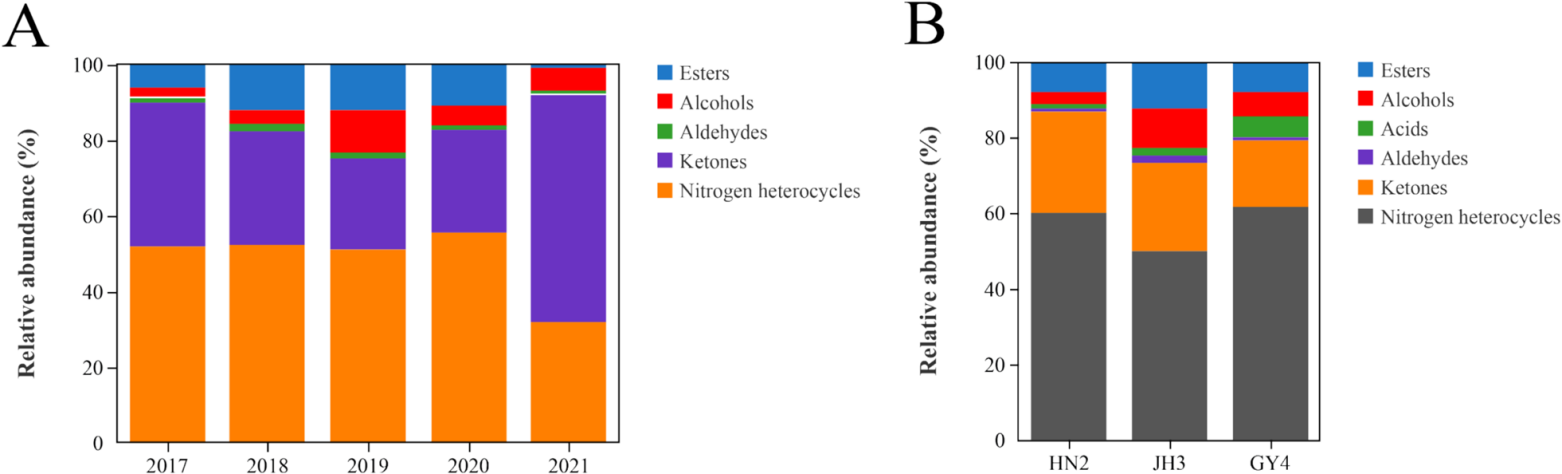
The aroma profile of cigar tobacco leaves. **A** Different years. **B** Different varieties

The primary constituents of different CTLs included nicotyrine, megastigmatrienone, and myosmine, with total proportions of 50.0–74.7%. The proportions of isonicoteine and dihydroactinidiolide in 2017–2020 were higher, reaching 11.0–12.5% and 5.69–11.0%, respectively, and geranyl acetone in 2017, 2020, and 2021 reached 16.1%, 7.59%, and 10.3%, respectively. The changes in the aroma proportion from different CTLs were similar to the content. For example, the proportions of esters, alcohols, aldehydes, and nitrogen heterocycles showed an increase first and then decrease with the increase of the year (Fig. 1A), and their proportions were 0.801–12.0%, 2.50–11.1%, 0.870–1.93%, and 32.0–55.5%, respectively, while the ketones were the opposite, and its proportion ranged from 23.9% to 60.2%. The aroma profile of 2021 CTL was significantly different from those of 2017–2020. Sun et al. (2011) reported that the acidic constituents increased, while the alkaline constituents and pH decreased during the aging of flue-cured tobacco. The microenvironment could be changed by the secondary metabolites in the tobacco aging process (Zhou et al. 2021). The microorganisms were beneficial for increasing and transforming the aroma substances during the tobacco leaves aging process (Zhou et al. 2020).

There were 24, 32, 30, 28, and 27 constituents detected in CTLs from 2017 to 2021, respectively. Sclareolide, safranal, dehydro-β-ionone, and 3-phenylpyridine were not detected, and 2-hexyldecanol and benzophenone were newly found in 2017 CTL, with contents of 29.0 μg/kg and 7.67 μg/kg, respectively. Methyl tetradecanoate, 2-hexyldecanol, and acetone were newly detected in 2018, with contents of 17.1 μg/kg, 5.38 μg/kg, and 16.2 μg/kg, respectively. Thunbergol and d,l-anatabine were not detected, and α-acetyl-α-methyl-γ-butyrolactone (15.4 μg/kg) and trans-*p*-menth-1-ol (28.6 μg/kg) were newly found in 2019. Hexanal and decanal were not detected, and methyl pentadecanoate (24.5 μg/kg) and methyl hexadecanoate (165 μg/kg) were newly found in 2020. Dihydroactinidiolide, cotinine, and isonicoteine were not detected, and 3-oxo-α-ionol (204 μg/kg) and hexadecanol (30.3 μg/kg) were newly found in 2021.

**Fig. 2:**
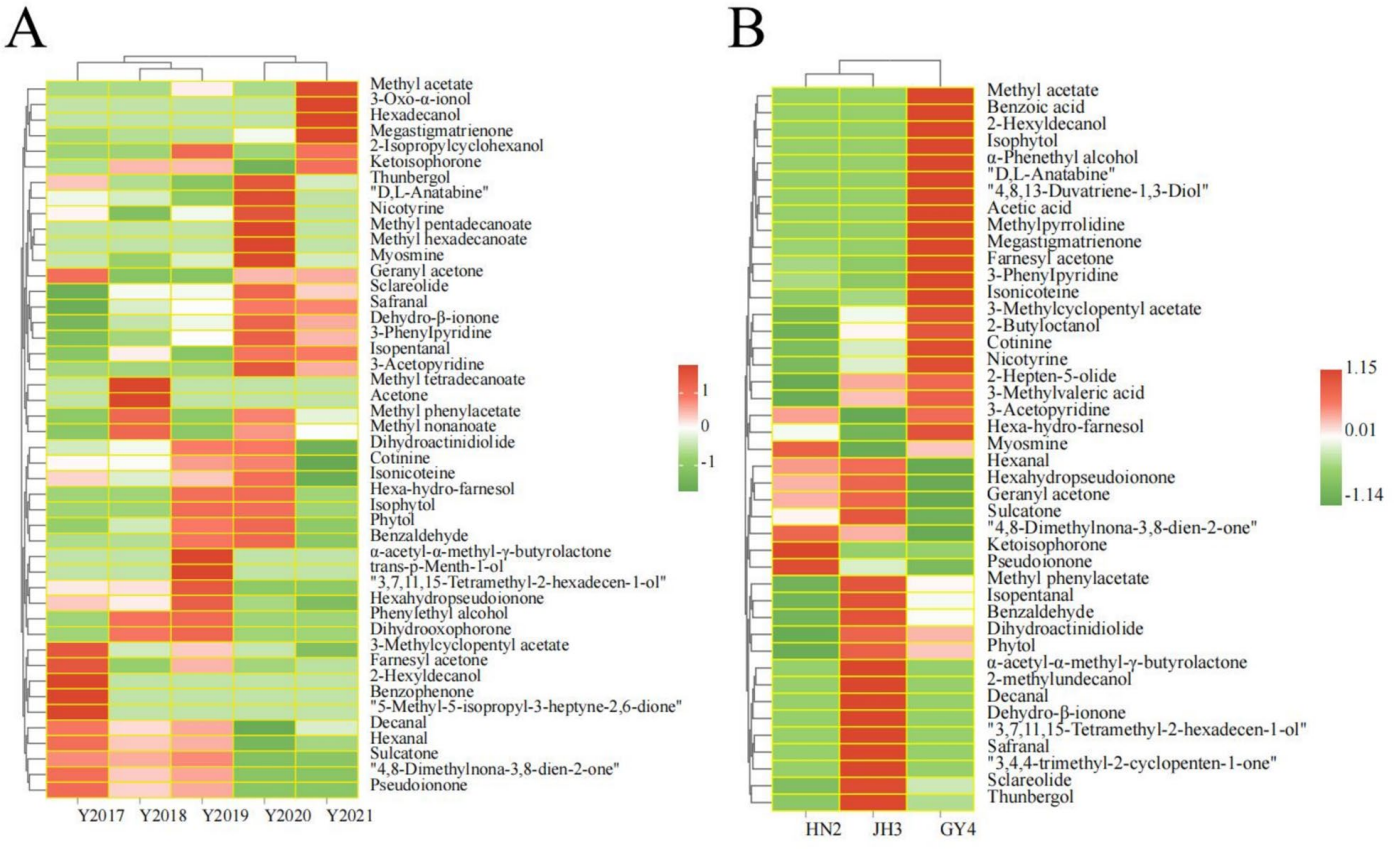
The heatmap of aroma constituents of cigar tobacco leaves. **A** Different years. **B** Different varieties

The contents of esters increased at first, subsequently decreasing with further increase of years, and the varieties of ester constituents detected from 2017 to 2021 were 2, 6, 5, 7, and 5, respectively. The changes in contents of sclareolide and dihydroactinidiolide with the years were the same as that of total esters (Fig. 2A), and the sum of the two constituents accounted for 93.2%, 85.1%, 94.9%, 72.4%, and 43.8% of the total esters content from 2017 to 2021, respectively. The total esters content (824 μg/kg) was the highest in 2020 CTL, due to the new detection of methyl pentadecanoate and methyl hexadecanoate, and methyl phenylacetate, dihydroactinidiolide, and sclareolide were significantly increased. Dihydroactinidiolide helps to mask bad flavors and enhance cigarette palatability, and many ester/lactone constituents contribute to fruity aromas (Pico et al. 2015). Contents of methyl nonanoate and methyl tetradecanoate significantly increased in 2018, and the contents of 3-methylcyclopentyl acetate, α-acetyl-α-methyl-γ-butyrolactone, and methyl acetate reached the highest in 2017, 2019, and 2021, respectively.

Four, 5, 7, 4, and 5 alcohol constituents were detected in CTLs from 2017 to 2021. 3-Oxo-α-ionol and hexadecanol were only detected in 2021 CTL, and 2-hexyldecanol was detected in 2017 and 2018 CTLs. The changes in the contents of phenylethyl alcohol, isophytol, phytol, and hexa-hydro-farnesol were similar to that of the total alcohols content, which increased at first, subsequently decreasing with a further increase over the years. The total alcohols content reached 561 μg/kg in 2019 CTL, and its content of trans-p-menth-1-ol, 2-isopropylcyclohexanol, and phenylethyl alcohol was significantly higher than in other years. There was a significant increase in the contents of hexa-hydro-farnesol, isophytol, and phytol in the 2019 and 2020 CTLs.

There were significant differences in total aldehydes and aldehyde constituents in CTLs from different years. The contents of total aldehydes and benzaldehyde enhanced at first, subsequently decreasing with further increase of years, and their contents increased significantly in 2019 CTL, reaching 97.1 μg/kg and 64.9 μg/kg, respectively. The contents of hexanal and decanal increased with the increase of years, their contents increased significantly in 2017 CTL, while the contents of isopentanal and safranal decreased, and their contents were higher in 2020 and 2021 CTLs.

For the CTLs from 2018 to 2021, the total ketones content decreased with the increase of the year. The total ketones content of 2017 CTL was lower than in 2021, but higher than that of 2018 and 2019, due to the megastigmatrienone content decreasing with the increase of the year, and geranyl acetone, pseudoionone, and benzophenone were newly detected in 2017. Acetone was only detected in the 2018 CTL. The contents of hexahydropseudoionone and dehydro-β-ionone increased at first, subsequently decreasing with further increase of years, their contents were the highest in 2019 and 2020, respectively. Among the CTLs from 2018 to 2021, the contents of sulcatone, dihydrooxophorone, 4,8-dimethylnona-3,8-dien-2-one, and pseudoionone were significantly higher in 2018 and 2019 CTLs than those in 2020 and 2021, and the farnesyl acetone content in 2019 was significantly higher than the others.

The d,l-anatabine content in 2020 CTL was higher than in other years, other nitrogen heterocycle constituents and total nitrogen heterocycles increased at first, subsequently decreasing with further increase of years, their concentrations reached the highest in 2020. Aroma profiles and characteristic aroma constituents were changed regularly, and aroma profiles induced such as esters, ketones, and nitrogen heterocycle were enriched after a specific aging period, and not always linearly dependent on the aging period, which was similar to the aroma profiles succession in other fermentation food during the aging process (Liu et al. 2025). This study revealed the variations of aroma constituents of CTLs over 5 years, which helps to understand the influence of aging periods on the aroma and contributes to the aroma and process regulation of CTLs.

### The aroma profile and differential constituent of CTLs from different varieties

There was also a significant difference in the aroma constituent contents from different CTL varieties. Forty-three aroma constituents were found in CTLs from three varieties, which included the esters (7), alcohols (10), acids (3), aldehydes (5), ketones (11), and nitrogen heterocycles (7), with the contents of 459–935 μg/kg, 172–803 μg/kg, 78.9–604 μg/kg, 43.6–142 μg/kg, 1.54–1.86 mg/kg, and 3.43–6.43 mg/kg, respectively, and the content of total aroma constituents was between 5.71 mg/kg and 10.5 mg/kg (Table S2). Among them, the aldehydes content was the highest in JH3 CTL, which was 2.25 and 1.19 times higher than HN2 and GY4, respectively. Ketones, nitrogen heterocycles, and acids reached the highest in GY4. Alcohols and esters of JH3 and GY4 were both higher than HN2. The distinct concentrations of these components confer unique olfactory characteristics to cigars, distinguishing them from other tobacco types.

The primary constituents of CTLs from different varieties included nicotyrine, isonicoteine, geranyl acetone, and myosmine, reaching 21.2–23.7%, 17.1–29.7%, 7.05–14.3%, and 7.16–13.9%, respectively. Dihydroactinidiolide, farnesyl acetone, 3-acetopyridine, megastigmatrienone, and 3-methylvaleric acid were all detected in three varieties, with total proportions of 18.0–20.1%. Thunbergol, phytol, and 2-hepten-5-olide were detected, which were 7.47%, 1.98%, and 1.09% in JH3, and were 1.03%, 1.04%, and 1.00% in GY4. The differences in the aroma profiles were small (Fig. 1B), and the ratios of ketones, nitrogen heterocycles, esters, alcohols, acids, and aldehydes in different varieties were 17.8–26.9%, 50.0–61.5%, 7.92–12.4%, 3.00–10.7%, 1.38–5.78%, and 0.620–1.89%, respectively.

Esters contribute sweet and fruity aromas to the tobacco profile. The total esters content of JH3 and GY4 CTLs increased by 1.04 and 0.80 times compared with HN2, and other ester constituents of HN2 also decreased significantly (Fig. 2B and Table S2). Methyl acetate and α-acetyl-α-methyl-γ-butyrolactone were only detected in JH3 and GY4 CTLs. The 3-methylcyclopentyl acetate content in GY4 CTL increased than other varieties, and the contents of methyl phenylacetate and sclareolide in JH3 increased significantly.

Alcohols play a role in amplifying floral and fruity aromas. The total alcohols content of JH3 and GY4 was 3.68 and 2.89 times higher than that of HN2, and other alcohol constituents of HN2 also decreased significantly. 2-Methylundecanol was only found in JH3 CTL, thunbergol content was higher than in other varieties. α-Phenethyl alcohol, 2-hexyldecanol, isophytol, 4,8,13-duvatriene-1,3-diol were newly detected, and the contents of hexa-hydro-farnesol and 2-butyloctanol of GY4 increased significantly than other varieties. Most alcohol components are formed by fatty acid metabolism using fatty acids as precursors, and different substrates and enzyme activities produce different alcohol aroma components, thus affecting tobacco aroma profiles. Besides, total acids of GY4 CTL enhanced by 3.40 and 6.66 times compared with HN2 and JH3, respectively, as acetic acid and benzoic acid were newly detected in GY4, and 3-methylvaleric acid content increased by 1.05 times compared with HN2. Volatile acidic constituents exhibit a significant influence on tobacco aroma and taste.

Aldehydes mainly originate from the unsaturated fatty acids oxidation or amino acid degradation, with a fatty flavor. C_2_–C_4_ aldehydes have a pungent odor such as pungent and fishy, while C_5_–C_9_ aldehydes have a green and wax flavor (Hou et al. 2017). Total aldehydes of JH3 CTL enhanced by 2.25 and 1.19 times compared with HN2 and GY4, respectively. Decanal and safranal were newly detected, and the latter was promoted to increase the sweetness, isopentanal and benzaldehyde were enhanced in JH3. Benzaldehyde has an almond aroma at lower concentrations, while higher concentrations give the tobacco a pleasant fruity aroma. Ketones are integral in forming key aromatic molecules via carotenoid degradation. The total ketones content of GY4 CTL increased significantly, reaching 1.86 mg/L, due to the contents of megastigmatrienone, cotinine, and farnesyl acetone being higher than HN2 and JH3. Megastigmatrienone primarily imparts woody and floral aromas to tobacco, and it is an important aroma component, the higher its content, the fuller the aroma (Wu et al. 2023b). Dehydro-β-ionone was newly detected in JH3 CTL, and sulcatone and hexahydropseudoionone were enhanced than other varieties. Ketoisophorone was newly detected, and 4,8-dimethylnona-3,8-dien-2-one content was increased in HN2.

Tobacco aroma contains a variety of nitrogen heterocycles, mainly pyridine, pyrrole, and pyrazine. Nitrogen heterocycles have a unique aromatic flavor, giving tobacco a strong roasted aroma, but also on the complexity of the tobacco aroma to produce a certain effect. The total nitrogen heterocycles content of GY4 increased significantly, due to methylpyrrolidine and d,l-anatabine being newly detected, and the contents of 3-phenylpyridine, nicotyrine, and isonicoteine were increased. There was no significant difference in the contents of nitrogen heterocycle constituents between HN2 and JH3. There were many varieties of CTLs, and each variety has its unique aroma profile. This study showed that the difference in the composition and the constituent contents may be an important factor for the different aromas of varieties.

### The microbial community composition and succession of CTLs from different aging periods

Effective sequence range for bacteria and fungi of CTLs from 5 years was 63,705–108,686 and 121,870–134,263 (Table S3), and the average ratio of the effective sequence was 80.9% and 93.5%, respectively. The effective sequence range of bacteria and fungi of CTLs from three varieties was 51,829–117,685 and 71,597–138,020, and the average ratio of the effective sequence was 83.7% and 89.4%, respectively. As the sequencing depth increased, the dilution curve gradually became flat (Figures S1 and S2), which indicated data could sufficiently represent diversity.

**Fig. 3:**
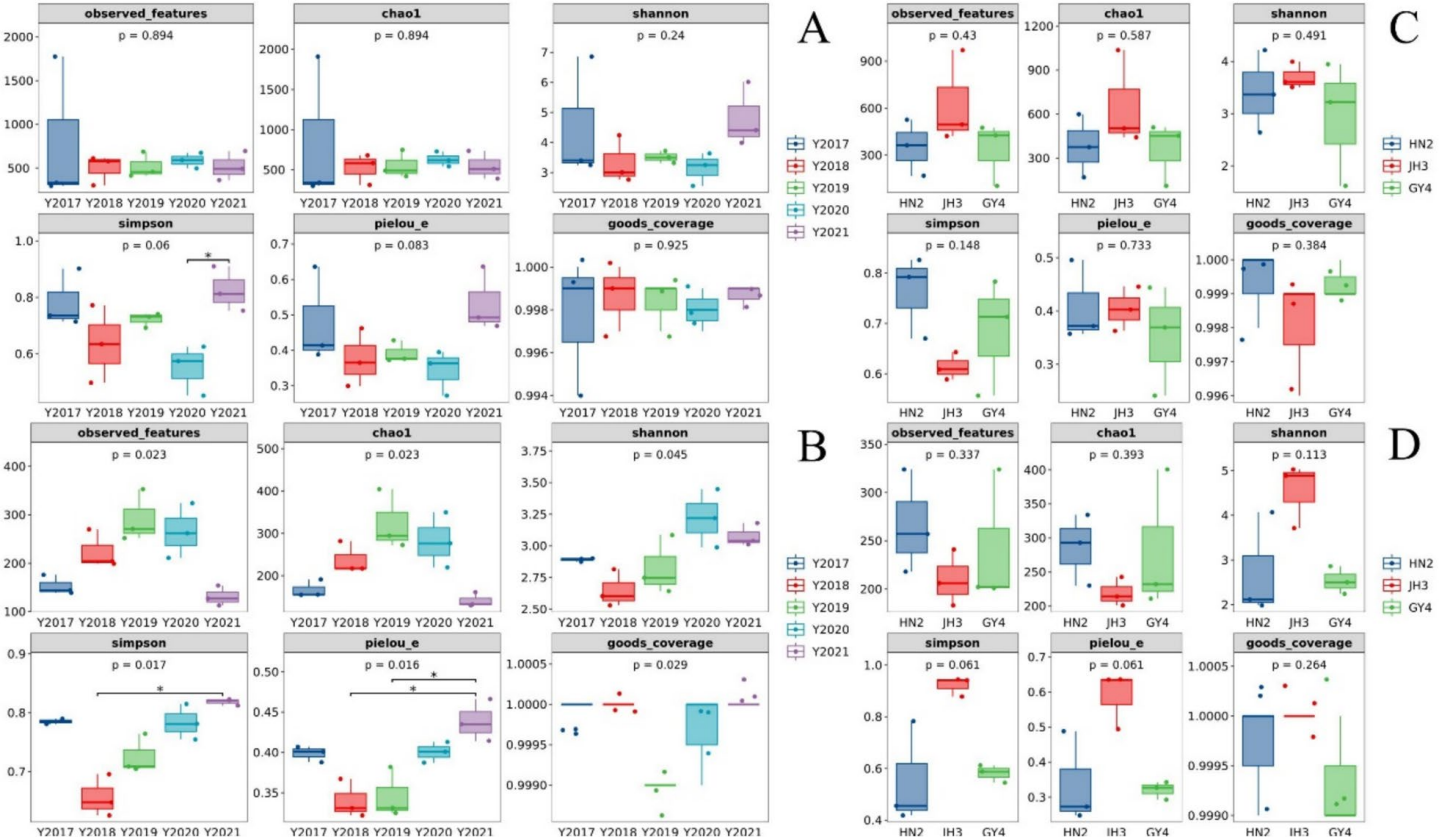
The microbial community α-diversity indices of cigar tobacco leaves from different years and varieties. **A** Bacterial community of different years. **B** Fungal community of different years. **C** Bacterial community of different varieties. **D** Fungal community of different varieties

**Fig. 4:**
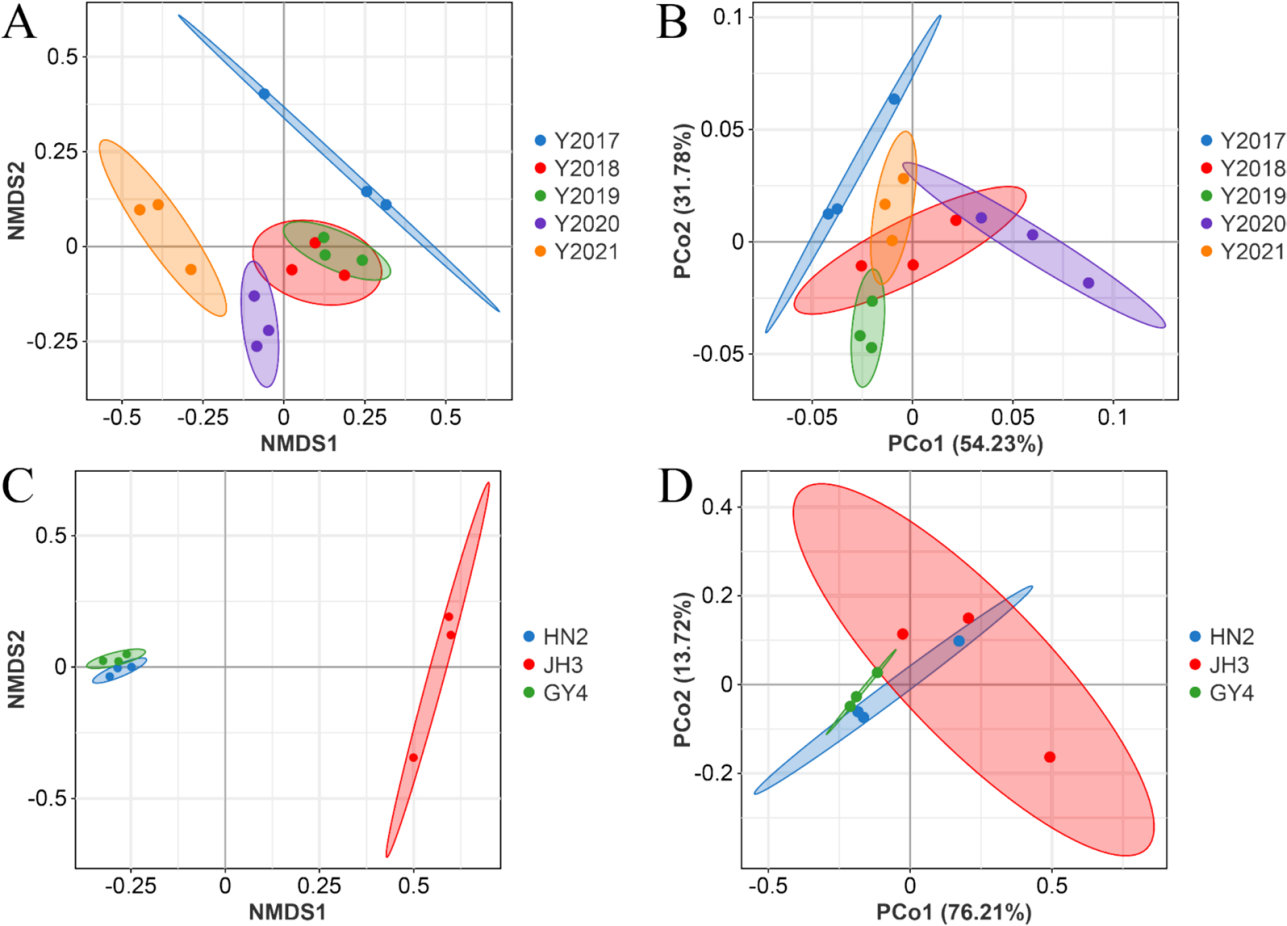
The microbial community β-diversity analysis of cigar tobacco leaves from different years and varieties (OTU > 1%). **A** Non-metric multidimensional scaling (NMDS) analysis of the bacterial community of different years. **B** Principal coordinate analysis (PCoA) of the fungal community of different years. **C** NMDS analysis of the bacterial community of different varieties. **D** PCoA of the fungal community of different varieties

The Chao1 index of the community increases when the abundance of species decreases. A high community diversity reflects the species are evenly distributed and the Shannon index is large. The Simpson index increases as the species evenness enhances. The Plelou’e evenness index of the fungal community of 2021 CTL was higher than that of 2018 and 2019, and the Simpson index of 2021 CTL was significantly higher than 2018 (Figs. 3A and B). The NMDS analysis illustrated that the bacterial communities of 2018 and 2019 CTLs were similar (Fig. 4A). These two CTLs were clearly distinguished from 2017, 2020, and 2021 CTLs, and there were also obvious differences between 2017, 2020, and 2021. PCoA showed that the fungal communities between 2017, 2019, 2020, and 2021 CTLs were different (Fig. 4B).

The bacterial communities of CTLs from five years included 46 phyla. *Cyanobacteria*, *Firmicutes*, and *Proteobacteria* were dominant, and their abundances were 38.7–69.0%, 8.70–30.5%, and 13.6–36.9%, respectively. The abundances of these dominant phyla were shifted over the years, while the changes were unfixed. *Proteobacteria* and *Firmicute*s were also predominant in flue-cured tobaccos, which related to carbon degradation (Wang et al. 2018).

**Fig. 5:**
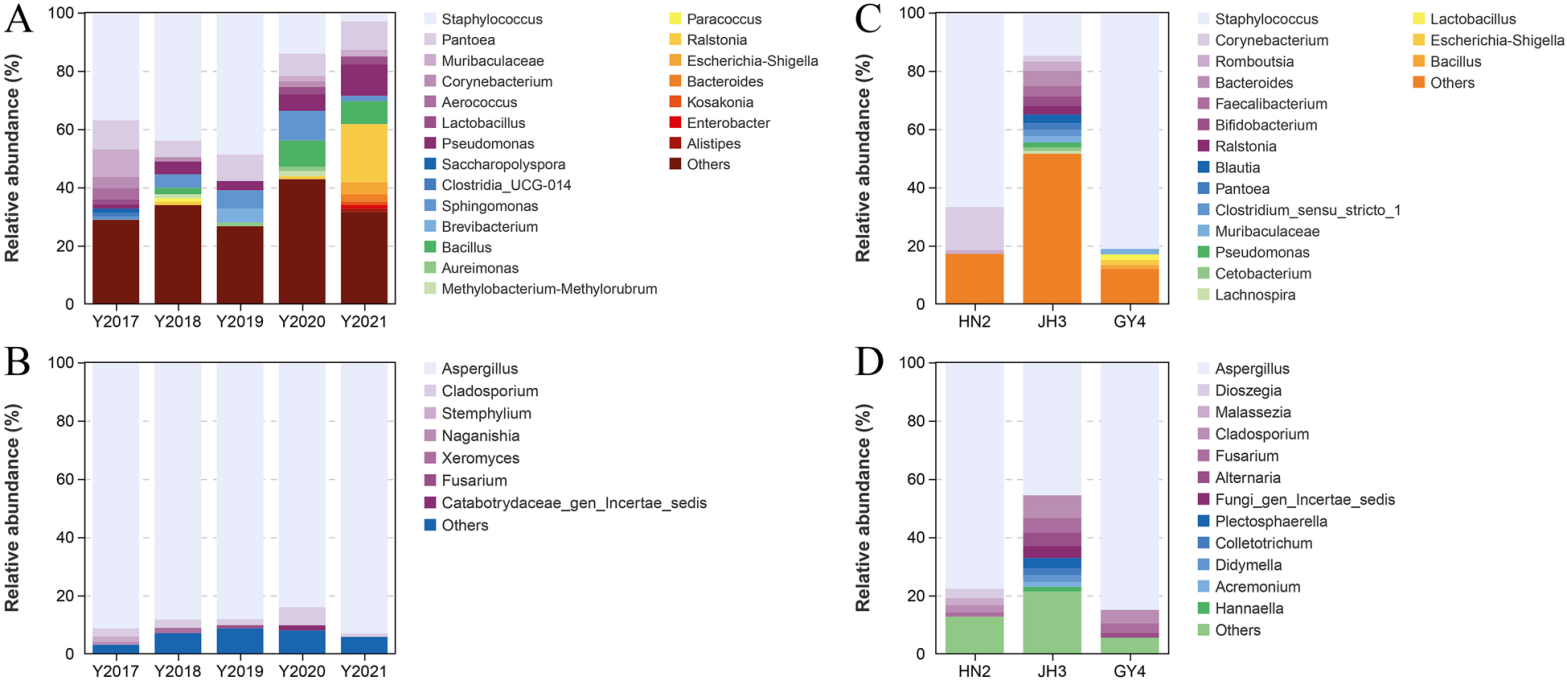
The microbial community composition of cigar tobacco leaves from different years and varieties (OTU > 1%). **A** Bacterial community of different years. **B** Fungal community of different years. **C** Bacterial community of different varieties. **D** Fungal community of different varieties

There were 915 genera belonging to 278 orders, the abundances of *Staphylococcus* (3.03–47.5%)*, Ralstonia* (0.669–20.2%), *Pseudomonas* (1.63–11.5%), *Pantoea* (6.07–10.5%)*,* and *Sphingomonas* (1.18–10.2%) were higher, and these communities showed specific succession with increasing years (Fig. 5A). Among them, *Staphylococcus* and *Sphingomonas* increased at first, subsequently decreasing with further increase of years, and their abundances were the highest in 2019 and 2020, respectively. *Staphylococcus* has been identified as a pivotal genus in flavor development due to its involvement in the catabolism of carbohydrates, lipids, and proteins, which contributed to the decomposition of branched-chain amino acids into other aroma compounds (Zhao et al. 2009). *Sphingomonas* exhibits significant metabolic activity in aromatic compounds, which includes sphingomyelin and phytosphingosine playing essential functions during tobacco fermentation (Li et al. 2020). But the abundances of *Pseudomonas*, *Ralstonia*, and *Pantoea* were the opposite and reached the highest in 2021, 2021, and 2017, respectively. *Pseudomonas* contributes to the catabolism of proteins and the enrichment of the amino acids, which plays a significant role in flavor development. Certain strains also possess the capability to degrade nicotine in tobacco, thus aiding in changing tobacco strength (Zhong et al. 2010). *Pantoea* exhibits strong resistance to environmental stress and is linked to different flavor constituents, with its viability being enhanced by indole derivatives produced by bacteria (Zheng et al. 2017). Additionally, *Bacillus* abundance in 2020 CTL was higher. This genus is capable of decomposing and utilizing macromolecular substances including starch, cellulose, and protein to generate aroma constituents, thereby promoting the tobacco flavor (Ye et al. 2024).

The fungal communities included 12 phyla, and *Ascomycota* was dominant with abundances of 95.2–97.7%. The fungal communities included 97 orders and 345 genera. *Aspergillus* and *Cladosporium* were all detected and their abundances were 84.0–93.0% and 1.19–6.22%, respectively (Fig. 5B), and their total abundances reached 90.2–94.2%. The dominant phylum and genera were consistent with those in flue-cured tobacco (Zhou et al. 2021). *Aspergillus* contributes to decomposition of sugars and proteins in tobacco, as well as biosynthesis of aroma constitutes. This genus is able to produce enzymes such as amylases and proteases, which facilitate formation of aroma constituents and the conversion of carbohydrates into aroma constituents, thereby enhancing the quality of cigars. *Cladosporium* produces enzymes that degrade macromolecules and produce flavor constituents (Zhang et al. 2021).

Similarly, obvious changes were in bacteria communities after six months of aging in tobacco leaves, while similarity in bacteria in 0 to 6 months of aging were observed (Zhao et al. 2007). The dominant bacteria during two years of aging of flue-cured tobacco included *Enterobacteriales* and *Pseudomonadales*, and fungi included *Incertae_sedis_Eurotiomycetes*, *Wallemiales*, and *Sporidiobolales,* and different from that in CTLs in our study, which may be due to difference in aging periods, origins, and raw materials (Zhou et al. 2020). Communities succession of bacterial and fungi during tobacco leaves aging process may primarily be determined by the total nitrogen and total organic carbon, respectively, which contributes to carbon organic constituent loss and increases the metabolites (Zhou et al. 2020). The fungal communities were related to cellulase activity, and the bacterial communities were strongly associated with amylase activity (Zhou et al. 2021).

Shortening aging time is important to tobacco industry, aiming to enhance productivity and product quality. Specific microbial communities contribute to accelerating chemical reactions in tobacco, such as certain bacteria, yeasts, and molds. Microorganisms can promote the degradation of proteins, carbohydrates, and lipids during tobacco aging to produce more volatile aroma constituents, which are important constituents of tobacco flavor. After aging tobacco treated with *Bacillus megaterium*, the content of most aroma constituents increased compared with that of naturally aged tobacco, and microbial agents effectively promote the degradation and transformation of tobacco constituents, shortening the aging period of tobacco by at least six months (Li 2008). Microorganisms also change chemical constituents and increase production rate of flavor constituents in tobacco by secreting enzymes and metabolites. The results of tobacco aging experiments with different varieties of flue-cured tobacco, different microorganisms and enzyme formulations, and different aging times, showed that the two different varieties of flue-cured tobacco significantly improved the intrinsic quality after two months of aging, which suggested that microorganisms and their enzymes were effective to shorten the natural aging period (Qian et al. 2006). Microbial fermentation can also reduce the content of alkaline constituents in the tobacco, so that the content of irritant constituents can be reduced, and the taste and flavor of the tobacco can be improved. The aging periods of CTLs were time-consuming, microorganisms improved flavor and shortened the aging periods. This study analyzed characteristics and differential microorganisms of CTLs, which provided a reference for screening functional microorganisms and shortening the aging periods of CTLs.

### The microbial community characteristics of CTLs from different varieties

There was no significant difference in the α-diversity index of microbial communities between different varieties of CTLs (Fig. 3C and D). NMDS analysis showed that the microbial communities between HN2 and GY4 CTLs were similar (Fig. 4C and D), while their communities were different from those of JH3.

The bacterial communities of CTLs from three varieties composed of 34 phyla, *Firmicutes was* dominant in HN2 and GY4 with abundances of 68.5% and 80.1%, respectively, while its abundance was decreased, and *Cyanobacteria* was dominant in JH3 (64.4%). There were 587 genera belonging to 193 orders. The dominant genus in HN2 was *Staphylococcus* (66.4%) and *Corynebacterium* (15.0%), and the former abundance in GY4 was 81.1% (Fig. 5C). The abundance of *Staphylococcus* in JH3 remarkably decreased, while the abundances of *Bacteroides*, *Faecalibacterium*, *Bifidobacterium*, *Romboutsia*, and *Ralstonia* increased. *Corynebacterium* is instrumental in producing aldehydes and ketones, which significantly enhance cigar flavor (Yao et al. 2022).

The fungal communities included 13 phyla, and *Ascomycota* was dominant, and its abundance in HN2, JH3, and GY4 was 87.0%, 79.2%, and 97.0%, respectively. There were 85 orders and 306 genera. The dominant fungi were all *Aspergillus* (Fig. 5D), their abundance in HN2, JH3, and GY4 was 77.7%, 45.6%, and 85.0%, respectively, while the abundances of *Cladosporium*, *Fusarium*, *Alternaria*, *Fungi*_*gen*_*Incertae*_*sedis*, and *Plectosphaerella* of JH3 were higher. *Alternaria* demonstrates the ability to degrade cellulose in CTLs, contributing to the biosynthesis of sugar derivatives (Macris 1984). The amylase of *Staphylococcus* and *Aspergillus* is primarily responsible for changes in total sugar content (Lakshmi et al. 2013). The predominant microorganisms exhibit capabilities in the degradation, transformation, metabolism, and utilization of matrix components within CTLs, thereby playing an important function in defining the quality attributes (Costa et al. 2020).

### Correlation of microbial communities with aroma constituents of CTLs from different aging periods

**Fig. 6:**
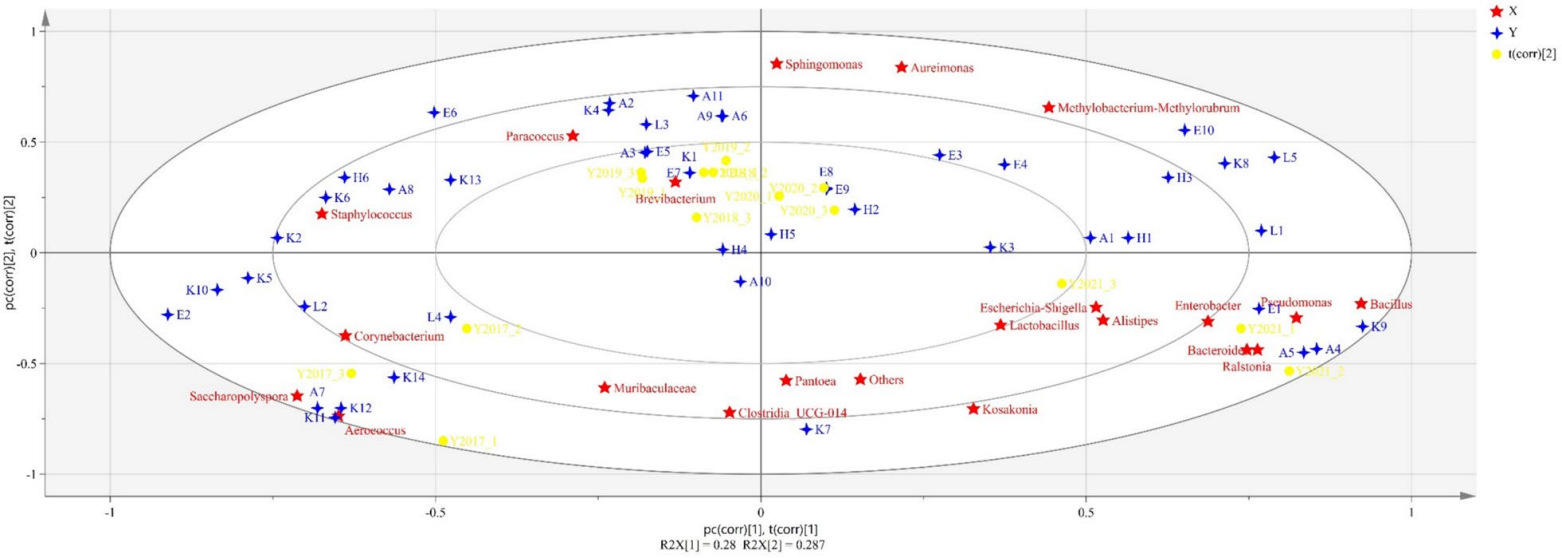
Partial least squares regression (PLSR) correlation of the aroma constituents against bacterial community in cigar tobacco leaves from different years (OTU > 1%). Aroma constituents used for the analysis are listed in Table S1

As the contribution of the fungal community to flavor is lower than bacterial community, bacterial genus was chosen for correlation analysis of the aroma profile. Based on the PLSR and VIP value analysis (Fig. 6), the differential bacteria (VIP > 1) between CTLs from different aging periods included *Staphylococcus*, *Paracoccus*, *Brevibacterium*, *Bacillus*, *Sphingomonas*, *Pseudomonas*, *Pantoea*, *Aureimonas*, *Saccharopolyspora, Methylobacterium-Methylorubrum*, and *Aerococcus*. Among them, *Staphylococcus* was linked to sulcatone, hexahydropseudoionone, and isonicoteine. *Paracoccus* was related to phenylethyl alcohol, benzaldehyde, and dihydrooxophorone. *Bacillus* was interrelated with megastigmatrienone. *Sphingomonas* was related to hexa-hydro-farnesol, isophytol, and thunbergol. *Methylobacterium-Methylorubrum* was connected to methyl nonanoate and sclareolide. *Aerococcus* was relevant to 5-methyl-5-isopropyl-3-heptyne-2,6-dione and benzophenone.

The functional microorganisms played an irreplaceable role in flavor enhancement and the attenuation of harmful substances in tobacco production. For example, co-inoculation of *Bacillus kochii* and *Bacillus amyloliquefaciens* showed enhanced concentrations of terpenoid and many Maillard reaction constituents than the others (Wu et al. 2021). *Filobasidium magnum*, *Bacillus kochii*, and *Bacillus amyloliticus* obtained from tobacco possessed capabilities of lipid oxidase, protein degradation, and starch degradation that enhanced tobacco fermentation effects (Wen et al. 2023). *Bacillus subtilis ZIM3* displayed high amylase and cellulase activities in tobacco, and the biodegradation function was enhanced (Dai et al. 2020).

## Conclusion

The contents of CTL aroma constituents were significantly affected by the aging periods and varieties. From 2017 to 2021, the contents of total aroma constituents, as well as proportions and contents of alcohols, esters, aldehydes, and nitrogen heterocycles increased at first, subsequently decreasing with further increase of years, while content and proportion of ketones were the opposite. The contents of total aroma constituents, esters, and nitrogen heterocycles increased significantly in 2020 CTL, alcohols and aldehydes enhanced in 2019, and ketones content was the highest in 2021. For the three varieties of CTLs, the aldehydes content was the highest in JH3, the contents of the total aroma constituents, acids, ketones, and nitrogen heterocycles were the highest in GY4, and the contents of esters and alcohols of the two varieties were significantly higher than HN2. Besides, the bacterial communities of CTLs from different years showed obvious community succession with increasing years. The abundances of *Staphylococcus* and *Sphingomonas* first increased and then reduced, their abundances were the highest in 2019 and 2020, respectively, while the abundances of *Pseudomonas*, *Ralstonia*, and *Pantoea* were the opposite, and their abundances were the highest in 2021, 2021, and 2017, respectively. The total abundances of *Aspergillus* and *Cladosporium* in fungal communities ranged from 90.2 to 94.2%. The dominant bacteria and fungi in CTLs of the three varieties were *Staphylococcus* and *Aspergillus*, respectively, their abundances in JH3 were significantly lower than those in HN2 and GY4. PLSR analysis showed that *Paracoccus*, *Staphylococcus*, *Aerococcus*, *Pantoea*, *Methylobacterium*-*Methylorubrum*, and *Bacillus* of CTLs from different years were related to aroma constituents.

## Supplementary Information

Supplementary Material 1.

## Data Availability

The datasets and materials used and/or analyzed during the current study are available from the corresponding author on reasonable request.
