# Supplementary material for: Characterization of aroma profile and microbial community of cigar tobacco leaves from different aging periods and varieties and their correlations analysis
Source: Bioresour Bioprocess. 2025 Jul 28;12(1):80. doi: 10.1186/s40643-025-00906-4 (PMC12304363; doi:10.1186/s40643-025-00906-4)
Supplement: Supplementary file 1 — Supplementary Material 1. [file 40643_2025_906_MOESM1_ESM.docx]

Table S1 The aroma constituents of cigar tobaccos leaves from different years (μg/kg)

| No. | RT | CAS | Constituents | Years | | | | |
| --- | --- | --- | --- | --- | --- | --- | --- | --- |
|  |  |  |  | 2017 | 2018 | 2019 | 2020 | 2021 |
|  |  |  | Esters (10) |  |  |  |  |  |
| E1 | 5.52 | 79-20-9 | Methyl acetate | ND | ND | 2.47±0.36^a^ | ND | 7.35±1.84^b^ |
| E2 | 13.25 | 24070-70-0 | 3-Methylcyclopentyl acetate | 20.27±1.52^c^ | 8.29±1.01^b^ | 12.87±1.23^d^ | 7.68±1.12^b^ | 2.71±0.19^a^ |
| E3 | 20.41 | 101-41-7 | Methyl phenylacetate | ND | 14.40±1.74^b^ | ND | 12.03±4.02^b^ | 5.31±0.60^a^ |
| E4 | 21.39 | 1731-84-6 | Methyl nonanoate | ND | 23.28±0.69^a^ | ND | 18.16±0.80^b^ | 11.26±0.28^c^ |
| E5 | 23.82 | 1123-19-9 | α-Acetyl-α-methyl-γ-butyrolactone | ND | ND | 15.35±0.90^a^ | ND | ND |
| E6 | 28.51 | 17092-92-1 | Dihydroactinidiolide | 278.85±53.45^a^ | 343.35±18.58^a^ | 556.41±85.83^b^ | 566.01±58.95^b^ | ND |
| E7 | 31.57 | 124-10-7 | Methyl tetradecanoate | ND | 17.06±1.46^a^ | ND | ND | ND |
| E8 | 33.18 | 7132-64-1 | Methyl pentadecanoate | ND | ND | ND | 24.49±4.76^a^ | ND |
| E9 | 34.38 | 112-39-0 | Methyl hexadecanoate | ND | ND | ND | 165.28±41.05^a^ | ND |
| E10 | 36.19 | 564-20-5 | Sclareolide | ND | 16.69±2.44^a^ | 16.59±1.09^a^ | 30.14±2.77^b^ | 20.75±3.27^a^ |
|  |  |  | Total | 299.12±53.25^a^ | 423.07±16.23^b^ | 603.68±86.97^c^ | 823.79±105.08^d^ | 47.38±4.22^e^ |
|  |  |  | Alcohols (11) |  |  |  |  |  |
| A1 | 18.58 | 96-07-1 | 2-Isopropylcyclohexanol | ND | ND | 95.49±4.13^a^ | ND | 87.52±6.67^b^ |
| A2 | 19.40 | 60-12-8 | Phenylethyl alcohol | ND | 10.70±0.37^a^ | 11.53±0.61^b^ | ND | ND |
| A3 | 22.93 | 3901-93-7 | trans-p-Menth-1-ol | ND | ND | 28.57±3.50^a^ | ND | ND |
| A4 | 30.34 | 34318-21-3 | 3-Oxo-α-ionol | ND | ND | ND | ND | 203.76±21.11^a^ |
| A5 | 30.50 | 36653-82-4 | Hexadecanol | ND | ND | ND | ND | 30.26±13.87^a^ |
| A6 | 31.65 | 6750-34-1 | Hexa-hydro-farnesol | ND | ND | 164.98±11.43^a^ | 168.97±20.61^a^ | ND |
| A7 | 31.76 | 2425-77-6 | 2-Hexyldecanol | 28.97±6.63^a^ | 5.38±0.77^b^ | ND | ND | ND |
| A8 | 33.68 | 102608-53-7 | 3,7,11,15-Tetramethyl-2-hexadecen-1-ol | 31.49±8.68^a^ | 32.64±4.98^a^ | 66.18±4.91^b^ | ND | ND |
| A9 | 34.61 | 505-32-8 | Isophytol | ND | ND | 26.57±2.48^a^ | 25.65±4.26^a^ | ND |
| A10 | 35.78 | 25269-17-4 | Thunbergol | 25.74±14.55^b^ | 8.80±1.60^ab^ | ND | 44.61±15.86^c^ | 13.56±1.91^ab^ |
| A11 | 36.08 | 150-86-7 | Phytol | 36.61±10.94^a^ | 75.33±9.53^b^ | 167.65±4.85^c^ | 186.59±39.78^c^ | 29.64±2.61^a^ |
|  |  |  | Total | 122.81±20.73^a^ | 132.86±16.19^a^ | 560.97±23.41^c^ | 425.82±70.14^b^ | 364.74±31.45^b^ |
|  |  |  | Aldehydes (5) |  |  |  |  |  |
| L1 | 7.25 | 590-86-3 | Isopentanal | ND | 1.58±0.40^a^ | ND | 2.70±0.37^b^ | 2.58±0.20^b^ |
| L2 | 10.58 | 66-25-1 | Hexanal | 12.21±0.49^c^ | 8.76±1.39^b^ | 9.56±0.23^b^ | ND | 3.41±0.81^a^ |
| L3 | 15.06 | 100-52-7 | Benzaldehyde | 35.24±3.81^a^ | 36.13±1.24^a^ | 64.90±9.54^b^ | 68.81±16.47^b^ | 27.36±7.61^a^ |
| L4 | 21.07 | 112-31-2 | Decanal | 21.05±3.48^c^ | 15.46±0.85^b^ | 18.06±1.07^bc^ | ND | 11.25±1.25^a^ |
| L5 | 21.12 | 116-26-7 | Safranal | ND | 3.75±0.46^a^ | 4.56±0.40^a^ | 7.02±1.48^b^ | 6.80±1.73^b^ |
|  |  |  | Total | 68.50±2.17^ab^ | 65.68±0.31^ab^ | 97.08±8.41^c^ | 78.53±14.72^b^ | 51.41±11.01^a^ |
|  |  |  | Ketones (14) |  |  |  |  |  |
| K1 | 5.23 | 67-64-1 | Acetone | ND | 16.16±0.58^a^ | ND | ND | ND |
| K2 | 15.45 | 110-93-0 | Sulcatone | 58.57±6.57^a^ | 54.17±2.43^a^ | 58.77±5.70^a^ | 18.39±7.16^b^ | 20.57±4.23^b^ |
| K3 | 19.70 | 1125-21-9 | Ketoisophorone | 9.49±1.51^ab^ | 11.65±1.02^bc^ | 11.61±0.42^bc^ | 7.67±1.46^a^ | 12.76±1.72^c^ |
| K4 | 20.31 | 20547-99-3 | Dihydrooxophorone | ND | 4.43±0.28^a^ | 5.07±1.76^a^ | ND | ND |
| K5 | 22.57 | 872858-42-9 | 4,8-Dimethylnona-3,8-dien-2-one | 26.95±6.77^b^ | 18.18±1.21^a^ | 21.07±3.43^ab^ | ND | ND |
| K6 | 25.49 | 1604-34-8 | Hexahydropseudoionone | 55.13±7.36^b^ | 52.88±1.01^b^ | 64.61±1.57^c^ | 44.52±3.64^a^ | 38.87±0.35^a^ |
| K7 | 26.44 | 689-67-8 | Geranyl acetone | 789.03±67.15^a^ | ND | ND | 581.92±55.66^b^ | 609.72±32.54^b^ |
| K8 | 27.16 | 1203-08-3 | Dehydro-β-ionone | ND | 37.80±6.20^a^ | 51.66±3.11^b^ | 104.74±8.93^c^ | 78.36±6.34^d^ |
| K9 | 28.82 | 38818-55-2 | Megastigmatrienone | 555.30±66.09^a^ | 656.50±97.05^a^ | 702.27±13.62^a^ | 1113.85±108.46^b^ | 2645.76±255.42^c^ |
| K10 | 29.10 | 141-10-6 | Pseudoionone | 45.97±6.81^a^ | 29.06±4.27^b^ | 34.27±2.86^b^ | ND | ND |
| K11 | 29.40 | 63922-44-1 | 5-Methyl-5-isopropyl-3-heptyne-2,6-dione | 48.05±7.92^a^ | ND | ND | ND | ND |
| K12 | 30.28 | 119-61-9 | Benzophenone | 7.67±0.95^a^ | ND | ND | ND | ND |
| K13 | 31.53 | 486-56-6 | Cotinine | 52.39±12.68^a^ | 50.27±7.38^a^ | 69.94±41.12^a^ | 75.10±54.94^a^ | ND |
| K14 | 34.29 | 1117-52-8 | Farnesyl acetone | 222.08±44.05^c^ | 138.12±26.20^a^ | 186.83±10.19^bc^ | 142.67±9.05^ab^ | 150.05±3.82^ab^ |
|  |  |  | Total | 1870.63±198.47^b^ | 1069.20±136.13^a^ | 1206.09±47.62^a^ | 2088.86±221.70^b^ | 3556.09±297.24^c^ |
|  |  |  | Nitrogen heterocycles (6) |  |  |  |  |  |
| H1 | 18.82 | 350-03-8 | 3-Acetopyridine | ND | ND | ND | 92.60±8.79^a^ | 53.14±12.13^b^ |
| H2 | 26.29 | 532-12-7 | Myosmine | 495.57±74.64^a^ | 349.85±23.94^a^ | 557.20±147.39^a^ | 1308.15±242.54^b^ | 536.90±81.35^a^ |
| H3 | 27.08 | 1008-88-4 | 3-Phenylpyridine | ND | 13.87±2.76^a^ | 33.67±7.80^b^ | 67.15±16.75^c^ | 45.61±5.13^b^ |
| H4 | 27.30 | 487-19-4 | Nicotyrine | 1398.33±34.77^b^ | 1032.02±49.80^a^ | 1350.81±188.98^b^ | 1806.89±126.47^c^ | 1233.48±67.99^b^ |
| H5 | 28.13 | 2743-90-0 | D,L-Anatabine | 42.58±10.22^b^ | 31.97±1.72^ab^ | ND | 134.12±41.14^c^ | 22.62±3.12^ab^ |
| H6 | 28.49 | 581-50-0 | Isonicoteine | 605.36±97.11^a^ | 434.79±30.56^a^ | 629.12±136.45^a^ | 844.98±165.52^b^ | ND |
|  |  |  | Total | 2541.84±208.12^b^ | 1862.50±94.61^a^ | 2570.80±478.65^b^ | 4253.89±510.69^c^ | 1891.75±147.60^a^ |
|  |  |  | Total aroma constituents | 4902.90±445.99^b^ | 3553.31±251.28^a^ | 5038.62±625.05^b^ | 7670.89±860.16^c^ | 5911.37±470.93^b^ |

Data are the means ± standard deviation (n=3)

Values in the same row with different letters indicate significant differences (*P* < 0.05) by Duncan’s tests

ND, not detected

Table S2 The aroma constituents of cigar tobaccos leaves from different varieties (μg/kg)

| No. | RT | CAS | Constituents | Varieties | | |
| --- | --- | --- | --- | --- | --- | --- |
|  |  |  |  | Hainan2 | Jianheng3 | Guyin4 |
|  |  |  | Esters (7) |  |  |  |
| E1 | 5.52 | 79-20-9 | Methyl acetate | ND | ND | 4.00±0.53^a^ |
| E2 | 13.25 | 24070-70-0 | 3-Methylcyclopentyl acetate | 29.37±1.13^a^ | 43.71±1.95^b^ | 61.24±7.90^c^ |
| E3 | 20.41 | 101-41-7 | Methyl phenylacetate | 11.09±0.71^a^ | 35.11±1.47^b^ | 23.73±2.31^c^ |
| E4 | 20.69 | 19895-35-3 | 2-Hepten-5-olide | ND | 82.23±25.87^a^ | 104.70±11.24^a^ |
| E5 | 23.82 | 1123-19-9 | α-Acetyl-α-methyl-γ-butyrolactone | ND | 47.02±5.30^a^ | ND |
| E6 | 28.51 | 17092-92-1 | Dihydroactinidiolide | 397.16±48.22^a^ | 676.75±131.19^b^ | 604.90±44.94^b^ |
| E7 | 36.19 | 564-20-5 | Sclareolide | 21.61±2.55^a^ | 49.98±14.56^b^ | 29.90±4.12^a^ |
|  |  |  | Total | 459.23±50.69^a^ | 934.80±175.03^b^ | 828.49±65.50^b^ |
|  |  |  | Alcohols (10) |  |  |  |
| A1 | 18.98 | 98-85-1 | α-Phenethyl alcohol | ND | ND | 4.97±1.22^a^ |
| A2 | 26.94 | 10522-26-6 | 2-Methylundecanol | ND | 29.54±5.42^a^ | ND |
| A3 | 31.65 | 6750-34-1 | Hexa-hydro-farnesol | 127.24±7.27^a^ | ND | 271.96±30.46^b^ |
| A4 | 31.76 | 2425-77-6 | 2-Hexyldecanol | ND | ND | 51.81±6.37^a^ |
| A5 | 33.68 | 102608-53-7 | 3,7,11,15-Tetramethyl-2-hexadecen-1-ol | ND | 55.63±9.50^a^ | ND |
| A6 | 34.61 | 505-32-8 | Isophytol | ND | ND | 26.75±4.09^a^ |
| A7 | 35.10 | 3913-02-8 | 2-Butyloctanol | ND | 6.45±1.36^a^ | 12.18±1.71^b^ |
| A8 | 35.78 | 25269-17-4 | Thunbergol | 22.76±3.90^a^ | 562.13±154.41^b^ | 107.87±22.50^a^ |
| A9 | 35.88 | 7220-78-2 | 4,8,13-Duvatriene-1,3-diol | ND | ND | 83.21±19.63^a^ |
| A10 | 36.08 | 150-86-7 | Phytol | 21.60±6.51^a^ | 148.84±30.62^b^ | 108.50±33.40^b^ |
|  |  |  | Total | 171.61±14.02^a^ | 802.60±141.04^b^ | 667.23±115.50^b^ |
|  |  |  | Acids (3) |  |  |  |
| C1 | 6.43 | 64-19-7 | Acetic acid | ND | ND | 180.39±69.16^a^ |
| C2 | 17.79 | 105-43-1 | 3-Methylvaleric acid | 78.90±24.61^a^ | 137.19±9.87^b^ | 161.67±11.25^b^ |
| C3 | 24.00 | 65-85-0 | Benzoic acid | ND | ND | 262.07±72.54^a^ |
|  |  |  | Total | 78.90±24.61^a^ | 137.19±9.87^a^ | 604.12±121.70^b^ |
|  |  |  | Aldehydes (5) |  |  |  |
| L1 | 7.25 | 590-86-3 | Isopentanal | ND | 7.19±0.56^a^ | 3.39±1.57^b^ |
| L2 | 10.58 | 66-25-1 | Hexanal | 5.65±0.99^a^ | 6.65±0.67^a^ | ND |
| L3 | 15.06 | 100-52-7 | Benzaldehyde | 37.98±6.97^a^ | 86.41±9.39^b^ | 61.39±10.42^c^ |
| L4 | 21.07 | 112-31-2 | Decanal | ND | 35.37±0.96^a^ | ND |
| L5 | 21.12 | 116-26-7 | Safranal | ND | 6.37±2.37^a^ | ND |
|  |  |  | Total | 43.63±6.00^a^ | 141.98±11.16^b^ | 64.77±11.15^c^ |
|  |  |  | Ketones (11) |  |  |  |
| K1 | 15.45 | 110-93-0 | Sulcatone | 27.72±2.14^a^ | 41.13±5.00^b^ | 12.19±1.25^c^ |
| K2 | 18.58 | 30434-65-2 | 3,4,4-Trimethyl-2-cyclopenten-1-one | ND | 147.39±13.66^a^ | ND |
| K3 | 19.70 | 1125-21-9 | Ketoisophorone | 5.26±0.67^a^ | ND | ND |
| K4 | 22.57 | 872858-42-9 | 4,8-Dimethylnona-3,8-dien-2-one | 31.88±2.94^a^ | 24.27±2.41^b^ | ND |
| K5 | 25.49 | 1604-34-8 | Hexahydropseudoionone | 50.42±2.67^a^ | 52.38±3.14^a^ | 44.35±2.03^b^ |
| K6 | 26.44 | 689-67-8 | Geranyl acetone | 815.89±22.22^a^ | 840.38±72.32^a^ | 737.45±41.60^a^ |
| K7 | 27.16 | 1203-08-3 | Dehydro-β-ionone | ND | 60.92±5.25^a^ | ND |
| K8 | 28.82 | 38818-55-2 | Megastigmatrienone | 270.09±29.07^a^ | 260.58±59.54^a^ | 558.86±49.21^b^ |
| K9 | 29.10 | 141-10-6 | Pseudoionone | 62.86±7.26^a^ | 50.99±8.88^ab^ | 44.60±4.69^b^ |
| K10 | 31.53 | 486-56-6 | Cotinine | 31.85±3.23^a^ | 46.84±7.06^a^ | 77.45±21.03^b^ |
| K11 | 34.29 | 1117-52-8 | Farnesyl acetone | 239.85±21.37^a^ | 222.85±64.27^a^ | 380.95±57.53^b^ |
|  |  |  | Total | 1535.81±78.51^a^ | 1747.74±214.65^b^ | 1855.85±159.04^c^ |
|  |  |  | Nitrogen heterocycles (7) |  |  |  |
| H1 | 7.93 | 120-94-5 | Methylpyrrolidine | ND | ND | 11.66±1.01^a^ |
| H2 | 18.82 | 350-03-8 | 3-Acetopyridine | 162.87±40.78^a^ | 130.73±16.12^a^ | 169.69±29.24^a^ |
| H3 | 26.29 | 532-12-7 | Myosmine | 792.82±29.72^a^ | 651.51±108.18^a^ | 748.37±72.34^a^ |
| H4 | 27.08 | 1008-88-4 | 3-PhenyIpyridine | 35.26±0.94^a^ | 32.00±9.47^a^ | 58.31±11.02^b^ |
| H5 | 27.30 | 487-19-4 | Nicotyrine | 1353.85±156.56^a^ | 1666.75±236.90^a^ | 2219.60±95.50^b^ |
| H6 | 28.13 | 2743-90-0 | D,L-Anatabine | ND | ND | 117.57±40.68^a^ |
| H7 | 28.49 | 581-50-0 | Isonicoteine | 1080.30±51.83^a^ | 1284.38±278.32^a^ | 3107.19±569.93^b^ |
|  |  |  | Total | 3425.09±234.50^a^ | 3765.37±644.00^a^ | 6432.39±723.31^b^ |
|  |  |  | Total aroma constituents | 5714.27±363.03^a^ | 7529.68±1168.47^a^ | 10452.86±1099.28^b^ |

Data are the means ± standard deviation (n=3)

Values in the same row with different letters indicate significant differences (*P* < 0.05) by Duncan’s tests

ND, not detected

Table S3 The sequencing result of microbial community of cigar tobaccos leaves from different years and varieties

| Samples | Effective Tags | | Paired-end reads | | Effective (%) | |
| --- | --- | --- | --- | --- | --- | --- |
|  | Bacteria | Fungi | Bacteria | Fungi | Bacteria | Fungi |
| Y2017.1 | 108686 | 127430 | 133923 | 135963 | 81.16% | 93.72% |
| Y2017.2 | 63705 | 129360 | 79113 | 136436 | 80.52% | 94.81% |
| Y2017.3 | 87552 | 125440 | 101234 | 132139 | 86.48% | 94.93% |
| Y2018.1 | 104769 | 129764 | 124098 | 135455 | 84.42% | 95.80% |
| Y2018.2 | 88195 | 130945 | 103791 | 135631 | 84.97% | 96.55% |
| Y2018.3 | 71007 | 127811 | 88764 | 133326 | 80.00% | 95.86% |
| Y2019.1 | 92296 | 126894 | 117796 | 133219 | 78.35% | 95.25% |
| Y2019.2 | 72094 | 133758 | 86433 | 144693 | 83.41% | 92.44% |
| Y2019.3 | 96092 | 134263 | 123458 | 143688 | 77.83% | 93.44% |
| Y2020.1 | 97201 | 123059 | 116347 | 136412 | 83.54% | 90.21% |
| Y2020.2 | 78949 | 121870 | 93435 | 135022 | 84.50% | 90.26% |
| Y2020.3 | 93345 | 124046 | 109876 | 135132 | 84.95% | 91.80% |
| Y2021.1 | 84577 | 124248 | 124727 | 134256 | 67.81% | 92.55% |
| Y2021.2 | 94686 | 124160 | 119894 | 133206 | 78.97% | 93.21% |
| Y2021.3 | 80110 | 124738 | 104018 | 135827 | 77.02% | 91.84% |
| HN2.1 | 84129 | 133893 | 111538 | 148277 | 75.43% | 90.30% |
| HN2.2 | 106608 | 129535 | 129234 | 142205 | 82.49% | 91.09% |
| HN2.3 | 51829 | 96705 | 63205 | 131597 | 82.00% | 73.49% |
| JH3.1 | 106289 | 130815 | 121123 | 140018 | 87.75% | 93.43% |
| JH3.2 | 117685 | 122510 | 132645 | 133473 | 88.72% | 91.79% |
| JH3.3 | 55840 | 71597 | 65763 | 82082 | 84.91% | 87.23% |
| GY4.1 | 96823 | 121242 | 126706 | 133081 | 76.42% | 91.10% |
| GY4.2 | 87854 | 135658 | 101199 | 144633 | 86.81% | 93.79% |
| GY4.3 | 76555 | 138020 | 86124 | 149401 | 88.89% | 92.38% |


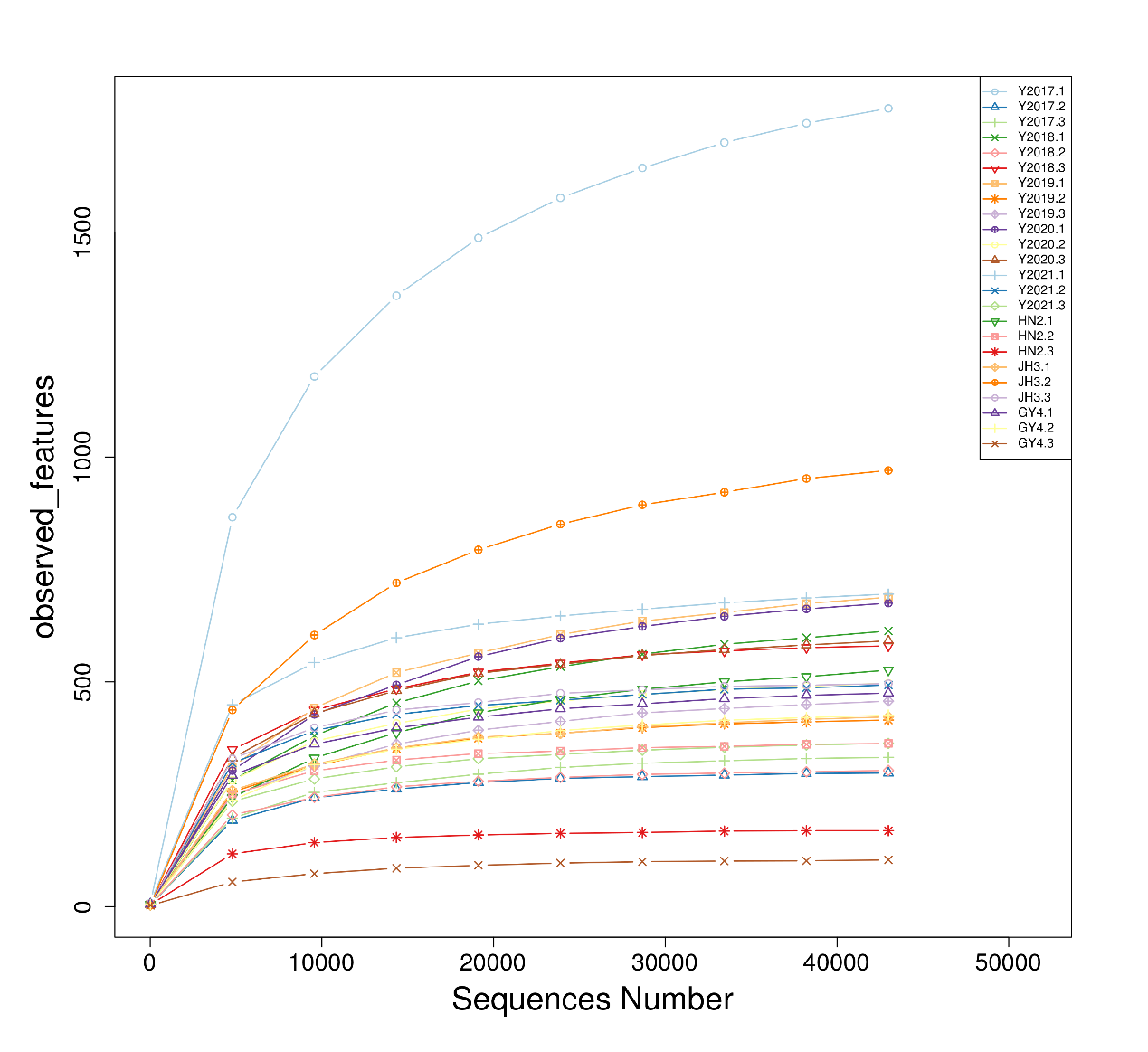


**Figure S1** Rarefaction curves of bacteria sequencing of microbial community of cigar tobaccos leaves from different years and varieties


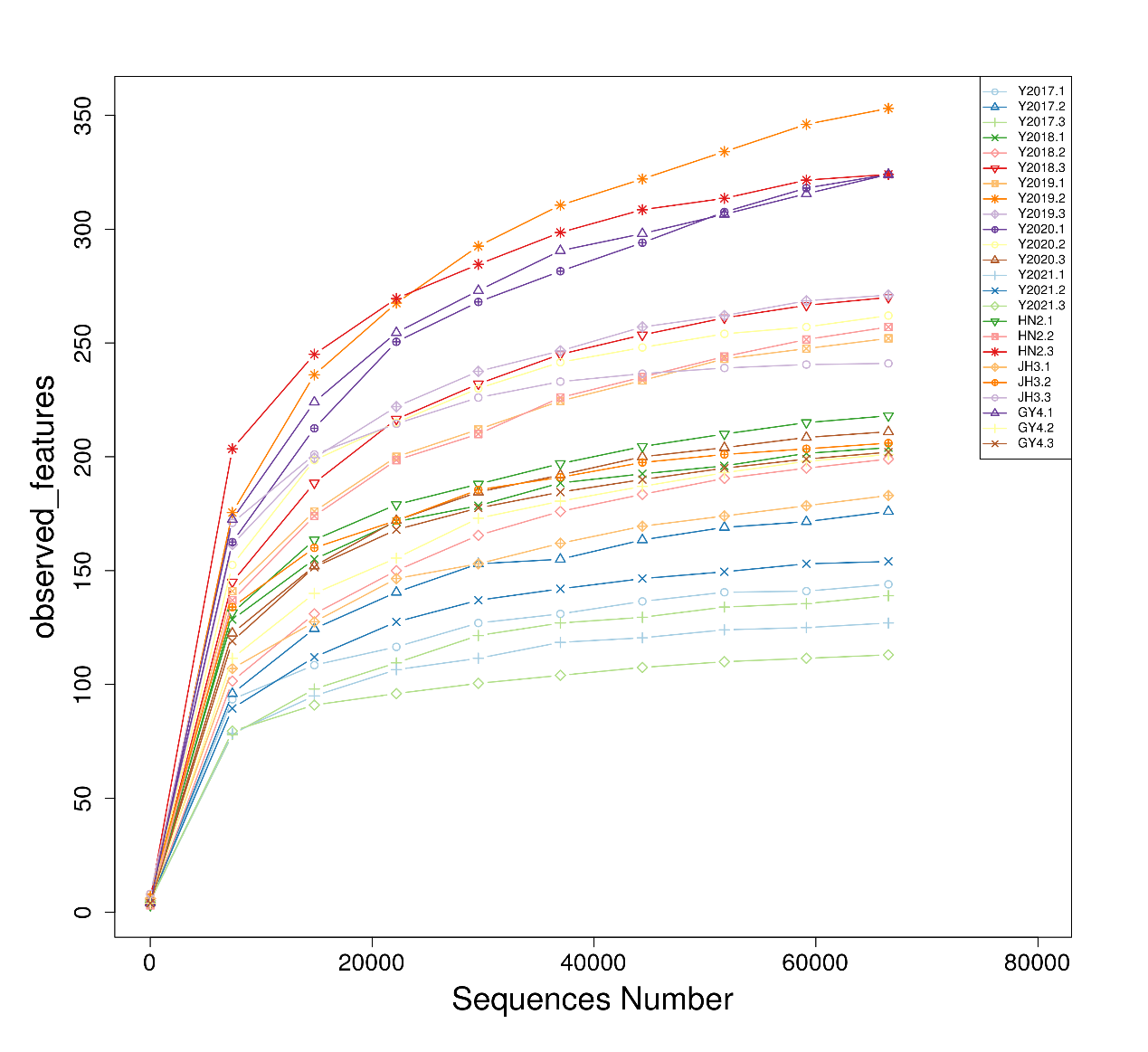


**Figure S2** Rarefaction curves of fungi sequencing of microbial community of cigar tobaccos leaves from different years and varieties
